# Can eye of origin serve as a deviant? Visual mismatch negativity from binocular rivalry

**DOI:** 10.3389/fnhum.2013.00190

**Published:** 2013-05-15

**Authors:** Manja van Rhijn, Urte Roeber, Robert P. O'Shea

**Affiliations:** ^1^Discipline of Psychology and Cognitive Neuroscience Research Cluster, School of Health and Human Sciences, Southern Cross UniversityCoffs Harbour, NSW, Australia; ^2^BioCog, Institute for Psychology, University of LeipzigLeipzig, Germany; ^3^Discipline of Biomedical Science, University of SydneySydney, Australia; ^4^Discipline of Psychology, School of Health and Human Sciences, Southern Cross UniversityCoffs Harbour, NSW, Australia

**Keywords:** visual mismatch negativity (vMMN), binocular rivalry, event-related potentials (ERP), attention, utrocular processing, eye-of-origin

## Abstract

The visual mismatch negativity (vMMN) is a negative deflection in an event-related potential (ERP) between 200 and 400 ms after onset of an infrequent stimulus in a sequence of frequent stimuli. Binocular rivalry occurs when one image is presented to one eye and a different image is presented to the other. Although the images in the two eyes are unchanging, perception alternates unpredictably between the two images for as long as one cares to look. Binocular rivalry, therefore, provides a useful test of whether the vMMN is produced by low levels of the visual system at which the images are processed, or by higher levels at which perception is mediated. To investigate whether a vMMN can be evoked during binocular rivalry, we showed 80% standards comprising a vertical grating to one eye and a horizontal grating to the other and 20% deviants, in which the gratings either swapped between the eyes (*eye-swap deviants*) or changed their orientations by 45° (*oblique deviants*). Fourteen participants observed the stimuli in 16, 4-min blocks. In eight consecutive blocks, participants recorded their experiences of rivalry by pressing keys—we call this the *attend-to-rivalry* condition. In the remaining eight consecutive blocks, participants performed a demanding task at fixation (a 2-back task), also by pressing keys—we call this the *reduced-attention* condition. We found deviance-related negativity from about 140 ms to about 220 ms after onset of a deviant. There were two noticeable troughs that we call an early vMMN (140–160 ms) and a late vMMN (200–220 ms). These were essentially similar for oblique deviants and eye-swap deviants. They were also essentially similar in the attend-to-rivalry conditions and the reduced-attention conditions. We also found a late, deviance-related negativity from about 270 to about 290 ms in the attend-to-rivalry conditions. We conclude that the vMMN can be evoked during the ever-changing perceptual changes of binocular rivalry and that it is sensitive to the eye of origin of binocular-rivalry stimuli. This is consistent with the vMMN's being produced by low levels of the visual system.

## Introduction

How do we process regularities and irregularities in our visual environments? The visual mismatch negativity (vMMN) is the electroencephalographic (EEG) signature of such processing (Czigler and Csibra, 1990). The vMMN arises when participants are exposed to a sequence of identical stimuli, called *standards*, in which every now and then, unpredictably, one of the standards is replaced by a stimulus, a *deviant* that differs in some way from the standards. As the name of the vMMN suggests, deviants yield event-related potentials (ERPs) that are more negative than those from standards.

Pazo-Alvarez et al. (2003) have reviewed studies of the vMMN. They found that deviants can be in the form, orientation, color, size, spatial frequency, and direction of movement of the stimuli. They defined the vMMN as occurring 250–400 ms after the onset of the deviant stimuli, beginning around the time of the second negative deflection in the ERP, the N2. Tales et al. (2009) have shown that the vMMN occurs when participants have withdrawn their attention from the stimuli [for a review, see Czigler (2007)], suggesting it is sign of a pre-attentive, automatic processing of irregularities in the visual environment.

The vMMN is thought to reflect processing that occurs when automatic predictions about upcoming stimuli are violated (Kimura et al., 2011). Based on the level of processing, Winkler and Czigler (2012) have argued that stimuli are represented as perceptual objects.

The phenomenon of binocular rivalry provides a test of the level of processing required for the vMMN. Binocular rivalry [e.g., reviewed by Blake and O'Shea (2009)] occurs when a person is presented with two different images, one to each eye (e.g., vertical lines to one eye and horizontal lines to the other). Instead of seeing a combination of the two images (i.e., a grid), the person sees one image for a second or so with no trace of the other, then the other image for a second or so with no trace of the first, then the first image, and so on, irregularly for as long as the person looks at the rival stimuli. Periods of exclusive visibility of one or the other image are usually separated by brief periods of some ever-changing mosaic or patchwork of the two images. All of this makes the conscious experience of binocular rivalry irregular and complex, yet the stimuli delivered to the eyes are unchanging. If the vMMN is an automatic, unconscious process, it should be possible to find it from a series of binocular-rivalry standards and deviants. However, if the vMMN requires attention—for the deviants to be experienced as rare and as different from the standards—then one would predict that the busy, ever-changing experience of binocular rivalry would banish the vMMN. It is this test we wanted to make.

**Figure 1:**
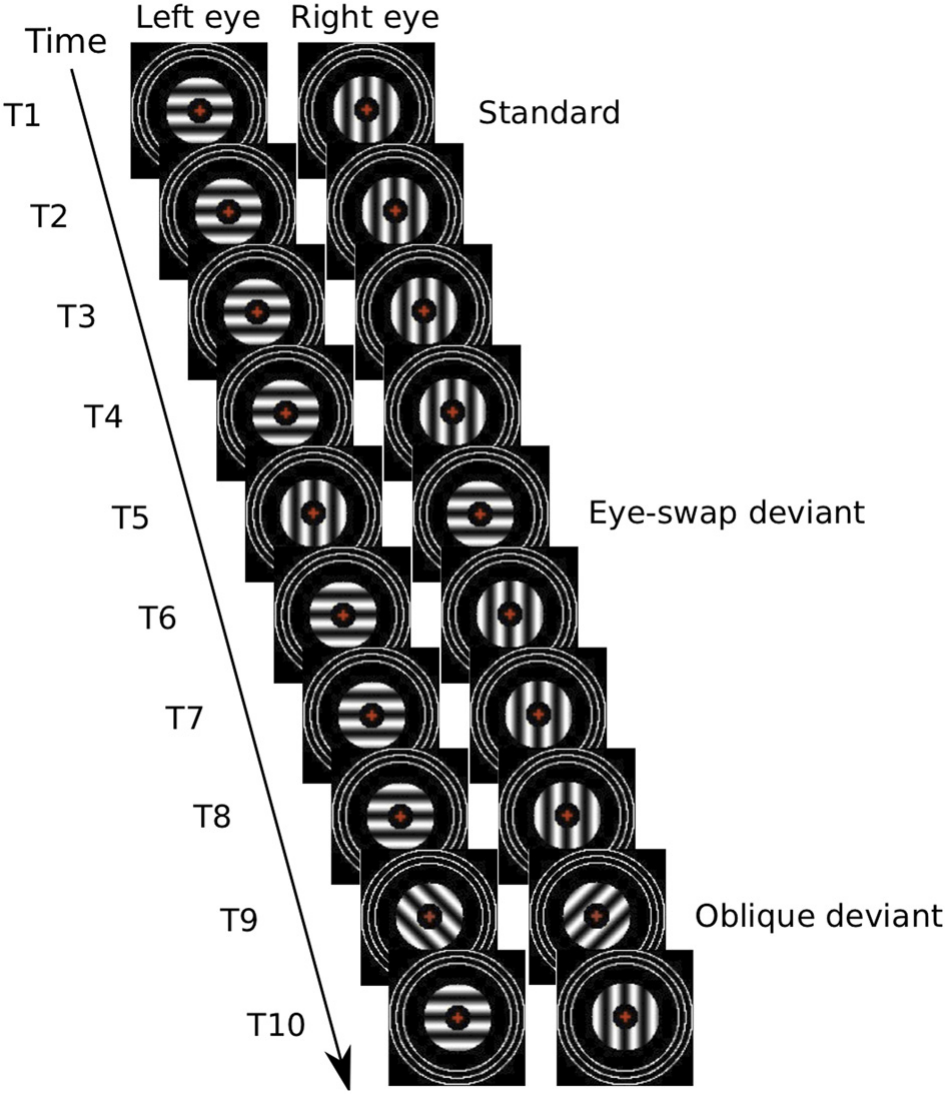
**Illustration of a possible sequence of 10 presentations of experimental stimuli.** In the first (T1), the left eye views a horizontal grating and the right eye views a vertical grating for 400 ± 33 ms, followed by no gratings for 100 ± 33 ms. This illustrates a standard; it is repeated for four presentations (i.e., T1–T4). The fifth presentation (T5) illustrates an eye-swap deviant. This is followed by three more standards followed by an oblique deviant (T9). Then there is a final standard (T10). The red cross in the center of the stimuli represents a red number that changed every 667 ms.

Our binocular rivalry standards were brief (400 ± 33 ms) displays of vertical lines to one eye and horizontal lines to the other (Figure 1). This time is easily enough for rivalry to be instigated and to develop into exclusive visibility of one or the other image (Wolfe, 1983; O'Shea and Crassini, 1984). Displays were separated by a briefer display (100 ± 33 ms) of a dark field. These are times that allows periods of exclusive visibility to persist over several displays of the rival stimuli (Noest et al., 2007; Klink et al., 2008).

We had two sorts of otherwise-identical, binocular rivalry deviants:

1. *Eye-swap* deviants: in these the orientations of both sets of lines were 90° clockwise from those of the standards. That is, the orientations were identical, but swapped between the eyes. It is possible that such deviants will not be perceptibly different from the standards. For example, Blake and Cormack (1979) have shown that participants cannot tell which eye is dominant during binocular rivalry. Moreover, regularly swapping the images between the two eyes at about 3 Hz has been reported to yield the usual experience of binocular rivalry, with periods of exclusive visibility of one of the images encompassing several eye swaps of the stimuli (Logothetis et al., 1996).
2. *Oblique* deviants: in these the orientations of both sets of lines was 45° clockwise from those of the standards. These deviants will be easily seen as different from the standards; they represent a control condition from which we expect a normal vMMN.

To test explicitly for the effects of attention on the vMMN, we ran two conditions, one in which participants had to pay attention to their conscious experience of the rivalry by pressing keys to report which of the rival stimuli they were seeing, and another in which they reduced any attention to the rival stimuli and paid attention to a demanding task (a 2-back task) in the center of the rival stimuli.

We found essentially identical vMMNs to both sorts of deviants. Reducing attention shortened the duration of the vMMN.

## Materials and methods

### Participants

Seventeen participants volunteered for this experiment. All participants where right handed and had normal or corrected-to-normal vision and visual acuity. All gave written, informed consent to participate and did so without any incentives, such as payment. The study was approved by Southern Cross University's Human Research Ethics Committee (approval number ECN-11-136).

One participant failed to experience binocular rivalry during a rivalry pre-test and so no other data were obtained from this participant. The data of two other participants were excluded because they did not yield enough epochs for at least one of the ERPs after data pre-processing (see below). Of the remaining 14 participants eight were female. Ages ranged from 21 to 58 years with a mean of 31.79.

### Apparatus and materials

Left-eye and right-eye stimuli were presented on the left and right sides of a linerarized, Samsung (2233RZ), 22-inch, color, LCD monitor (1680 × 1050 pixels; running at 60 Hz). Participants viewed stimuli from 57 cm through a Screenscope SA-200-Monitor-type, four, front-surfaced mirror stereoscope, attached to a chin rest. One participant opted to cross fuse the stimuli rather than using the stereoscope (he showed the same pattern of results as the other participants). Participants used a numeric keypad to respond. The experiment was run using a Macintosh Mini. This computer was controlled by custom-written MATLAB scripts using the Psychophysics Toolbox (Brainard, 1997; Pelli, 1997).

Electroencephalography (EEG) data were recorded continuously with a BrainAmp system (Brain Products GmbH, Munich) running on a Dell PC.

### Stimuli

There were three basic sorts of stimuli: grating stimuli, fusion stimuli, and fixation stimuli. *Grating stimuli* consisted of an annulus-shaped patch of achromatic, sine-wave grating shown to one eye and an orthogonal, but otherwise identical patch shown to the other eye. The outer diameter of a patch was 1.65° of visual angle; the inner diameter was 0.67°. Spatial frequency was 3.50 cycles/°, mean luminance was 43.37 cd/m^2^, and contrast was 0.99. They were displayed on a dark background (0.40 cd/m^2^).

A *fixation stimulus* was confined in the central region of the grating stimuli. It comprised of a central, red, one-digit number that changed every 667 ms to another randomly chosen number. The font style was Courier size 18 (0.50° height, ca. 0.30° width) with a pen width of 0.08°. These stimuli were identical in the two eyes.

*Fusion* stimuli were three, continuously presented, concentric, white (86.68 cd/m^2^), one-pixel-thick rings with diameters such that the smallest one was 50 min of visual angle larger than that of a grating. The diameter of the outer ring was 3.20° and had an even space of 0.10° cm between rings with a pen width of 0.05°. The fusion stimuli were identical to the two eyes. The fixation and fusion stimuli served to keep the eyes fixated centrally and aligned binocularly.

To form *rival stimuli*, one grating stimulus was shown to one eye and an orthogonally orientated grating stimulus was shown to the other, along with the fixation and fusion stimuli shown to both eyes (Figure 1). Some rival stimuli were *standards*; these had one arrangement of gratings to the eyes [e.g., left-eye horizontal (LEH) and right-eye vertical (REV)]. The remaining rival stimuli were *deviants*. There were two sorts: *eye-swap deviants* had the opposite arrangement of gratings to the eyes from the standards (i.e., LEV and REH) and *oblique deviants* had different orientations (e.g., left-eye, left oblique [LELO] and right-eye, right-oblique [RERO]). All rival stimuli had two combinations, one in which the stimuli were presented to the eyes as specified and one in which the stimuli were interchanged between the eyes.

Different stimuli were used to test visual evoked potentials (VEPs). The stimuli consisted of a central, 10-by-10 chequerboard, viewed on a gray background (43.37 cd/m^2^), with checks of 0.50° that phase reversed every 0.5 s for 50 s. At the same time, central red fixation numbers changed randomly every 667 ms.

### Procedure

We recorded the participant's sex, age, occupation, and dominant eye/hand. We measured the visual acuity of each participant's left eye, right eye, and both eyes together using the Freiburg Visual Acuity Test (Bach, 2007) at a viewing distance of 3.25 meters.

Then each participant responded in a rivalry pre-test. The participant viewed for 3 min binocular rivalry stimuli that were identical to the experimental stimuli except that there no deviants and pressed one key whenever and for as long as the vertical bars were visible with no trace of horizontal, and another key whenever and for as long as the horizontal bars were visible with no trace of vertical. The only difference from the standard stimuli in the experiment proper was that there was a continuously presented fixation cross instead of a changing fixation number. The first pre-test trial was then repeated with the opposite eye-orientation combination; order was counterbalanced.

Once the EEG electrodes were attached, we measured each participant's VEPs. The participant's task was to press a key when the fixation number was the same as the second last number shown. These VEP stimuli were presented once to the left eye while the right eye viewed the gray background, once to the right eye with gray to the left, and once to both eyes. Then they were repeated in the reverse order. Normal VEPs were defined as the VEPs' showing a N75, a P100, and a N135 that did not differ markedly between the eyes and that were larger for binocular stimulation (Odom et al., 2010; O'Shea et al., 2010). All participants showed normal VEPs.

The experiment proper consisted of 16 blocks. Each block involved 480 consecutive trials comprising 80% (384) standards, 10% (48) eye-swap deviants, and 10% (48) oblique deviants. Each trial was a display of rival stimuli for 400 ms with a uniform random jitter of ±33 ms, followed by the dark background for 100 ms with a uniform random jitter of ±33 ms. Order of trials within each block was randomized afresh for each participant and for each block with the constraints the first three and last two trials of each block had to show standard stimuli and that at least two standard-stimuli trials had to follow each deviant. Orientation-eye arrangement of standard rivalry stimuli alternated between blocks. Orientation-eye arrangement in the first block was counterbalanced across participants.

There were two attention conditions:

1. In the *attend-to-rivalry* condition, participants paid attention to the rival stimuli. We asked them to press one key whenever and for as long as they could see only horizontal lines and another key whenever and for as long as they could see only vertical lines, as they had done in the rivalry pre-test. This resulted in two events: a key press at the beginning of reporting an episode of dominance of one rival stimulus and a key release at the end. If participants saw anything else we asked them not to press either key. There were eight blocks in this condition.
2. In the *reduced-attention* condition, participants reduced their attention to the rival stimuli and devoted most, if not all, of their attention to the changing numbers at the fixation point. We asked them to press a key when the fixation number was the same as the second last shown—a 2-back task. The 2-back stimuli were presented in a randomized continuous stream with no repetitions and no interleaved targets. At the end of each block, the participant received feedback on the number of correct responses and on the number of false alarms in the 2-back task. There were eight blocks in this condition.

The numbers at fixation that changed every 667 ms to another randomly chosen number appeared in both conditions. Starting condition was counterbalanced over participants. In both conditions the participant was told to minimize eye blinks, and to relax.

### Measurement of EEG

EEGs were recorded from 26 active Ag/AgCl electrodes (F7, F3, Fz, F4, F8, FC5, FC1, FCz, AFz, FC2, FC6, T7, C3, Cz, C4, T8, CP5, CP1, CP2, CP6, P7, P3, Pz, P4, P8, O1, Oz, O2) mounted on an elastic cap (actiCap) placed according to 10–20 system and referenced to FCz, with the ground at AFz. The sampling rate was 500 Hz. A vertical electrooculogram (EOG) was recorded by electrodes above and below the right eye; a horizontal EOG was recorded by placing electrodes near the outer canthi of the eyes. Additionally an electrode was attached to each earlobe.

### Data analysis

#### Behavioral data

From the rivalry pre-test, we determined the mean time of episodes of dominance of one or the other rival stimuli. In the attend-to-rivalry condition, we determined the frequency and response time (RTs) of a key release from 150 to 1500 ms after the onset of a deviant stimulus. These measures let us know whether the deviants were perceived.

In the reduced-attention condition we determined detection and false alarm rates, from which we calculated sensitivities (*d*'), and the RTs for correct responses. These measures let us know whether the participants paid attention to the 2-back task rather than to the rival gratings.

#### Electrophysiological data

In preparation for data analysis, we re-referenced the EEG data offline to the right earlobe and applied a 0.5–35 Hz bandpass filter (Kaiser windowed sinc FIR filter, 1857 points). We extracted epochs of the data from 100 ms before to 400 ms after stimulus (gratings) onset. We excluded from further analysis any epochs preceding, containing, or following a key press within 300 ms. We also excluded any epochs with signals exceeding a moving-window, peak-to-peak amplitude of 200 μV at any EEG channel, or of 100 μV at any EOG channel (moving window width: 200 ms, distance between successive windows: 50 ms). Five data sets contained bad channels, which we corrected using spherical interpolation. The maximum number of channels we interpolated per data set was three. None of the channels was used in the statistical analysis.

We averaged ERPs separately for each stimulus type (standard, eye-swap deviant, oblique deviant) and condition (attention, reduced attention). We then excluded from further analysis two data sets that contained fewer than 100 epochs in any of the ERPs.

To investigate deviance-related differences we formed difference waves by subtracting the ERP to the standard stimuli from the ERPs to either of the deviant stimuli in both conditions. After visual inspection of the data for deviance-related differences, we defined three time windows of interest in each attention condition. Two of the time windows were the same for both attention conditions. Within these time windows we analysed the difference waves at occipital electrodes O1 and O2. We chose occipital electrodes for our analysis because gratings yield most pronounced responses in those electrodes.

We also calculated voltage maps for the various time windows to show the pattern of activity over all electrodes.

## Results and discussion

### Behavioral data

#### Rivalry pre-test

The mean duration of episodes of dominance of one or the other rival stimuli was 2086 ms (we give the standard deviation, SD, in parentheses after each mean, in this case 990 ms). The distributions of times showed pronounced positive skew. All this is consistent with rivalry reported by others (Fox and Herrmann, 1967; Levelt, 1967; Cogan, 1973; Zhou et al., 2004). That is, rivalry produced an ever-changing, unpredictable, sequence of percepts from which no regularity could be discerned.

#### Attend-to-rivalry condition

The mean duration of episodes of dominance of one or the other rival stimuli was 1567 ms (630 ms). The distributions of times showed pronounced positive skew. The general pattern is consistent with rivalry reported by others. The distribution was also bimodal. There was an early, sharp peak, between 600 and 700 ms, and a later, broader peak around 1200 ms. The early peak is likely due the episodes of dominance that were terminated by the occurrence of a deviant (see below); the later peak is likely due to naturally occurring rivalry alternations.

About 20% (12%) of all eye-swap deviants had no preceding key press, meaning that participants were experiencing some form of patchy dominance or combination of the rival images. Of the remaining trials, 74% (18%) resulted in a key release between 150 and 1500 ms after onset of the deviant. RTs were 691 (90) ms. That is, participants noticed the eye-swap deviants.

About 15% (9%) of all oblique deviants had no preceding key press. This difference from 20% for the eye-swap deviants must arise from sampling error, because oblique and eye-swap deviant were presented at random. Of the remaining oblique-deviant trials, 78% (13%) resulted in a key release between 150 and 1500 ms after onset of the deviant. This is not significantly different from the percentage of key releases for eye-swap deviants. RTs were 691 (56) ms—not significantly different from that for eye-swap deviants. That is, participants equally noticed both sorts of deviants.

We repeated these analyses with maximum window durations of 1000 and 650 ms. Apart from reducing the number of key releases and shortening the RTs, we found no significant differences for these measures from oblique deviants and from eye-swap deviants.

#### Reduced-attention condition

We defined a 2-back target as being detected when the participant pressed the key between 150 and 1000 ms after its occurrence. Participants detected on average (standard deviation) 49% (20%) of the 2-back targets. False alarm rate was 1% (0.7%). Mean *d*' was 2.23 (0.65). Participants' correct responses had an RT of 663 (72) ms. These results show that participants performed the 2-back task quite well but far from perfectly, suggesting that the task was demanding and occupied most, if not all, of their attention.

We did not ask participants if they were aware of the rivalry alternations during the reduced-attention condition. (Neither, for that matter, did we ask participants if they were aware of the fixation numbers, or of 2-back targets, during the attend-to-rivalry condition.) However, it is likely that participants noticed some rivalry alternations, especially if they had paid attention to rivalry in their first eight blocks. All we can really say are our own impressions from pilot testing: we felt that the 2-back task occupied our attention completely, however, occasionally we would notice a rivalry alternation, especially if it were abrupt. It was as if such alternations engaged attention exogenously.

### EEG data

On average there were 1264 (418) accepted epochs per participant for standard stimuli, 228 (82) for eye-swap deviants, and 225 (79) for oblique deviants in the attend-to-rivalry condition, and 1917 (178) accepted epochs for standard stimuli, 323 (28) for eye-swap deviants, and 323 (29) for oblique deviants in the reduced-attention condition.

**Figure 2:**
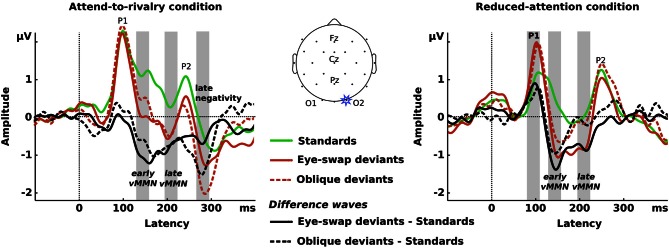
**Left panel:** ERPs (colored traces) and difference waves (black traces) from electrode O2 for the attend-to-rivalry condition. The gray vertical rectangles show the time windows for which we analysed the data. **Center panel:** Representation of the electrode array on a schematic head. **Right panel:** Same as the left panel for the reduced-attention condition. In the left panel, there is a clear negativity visible in the difference waves from about 140 ms after onset to about 350 ms. Difference waves from the two sorts of deviants are similar. In the right panel, there is a clear negativity visible in the difference waves from about 140 ms after onset to about 250 ms.

Figure 2 displays grand-averaged ERPs elicited by standard stimuli, by eye-swap deviants, and by oblique deviants and their difference waves (eye-swap deviants minus standards, oblique deviants minus standards) at the right hemisphere (O2) for both conditions separately. Activity was largest at electrodes O1 and O2 within all-time windows of interest. Data for the analyses were mean voltages across each time window and electrode.

The ERPs in both conditions show a similar pattern of deflections, starting with a pronounced positivity at about 100 ms (P1), a negativity at about 170 ms (N1), and a second positivity at about 250 ms (P2).

In both conditions, the earliest deviance-related negativity occurs at about 140 ms. Although this is earlier than Pazo-Alvarez et al. (2003) defined as being the vMMN, it is similar to results found by others for orientation changes in gratings (e.g., Winkler et al., 2005; Astikainen et al., 2008; Kimura et al., 2010). Certainly it is a deviance-related negativity.

In the attend-to-rivalry condition, this negativity sustains until about 350 ms with a second trough at about 280 ms for both types of deviants. In the reduced-attention condition, this earliest negativity sustains until about 250 ms for eye-swap deviants with another trough at about 200 ms, whereas it sustains only until about 170 ms for oblique deviants. For both eye-swap and oblique deviants in the reduced-attention condition we also see a deviance-related positivity at P1 that does not occur in the attend-to-rivalry condition.

**Figure 3:**
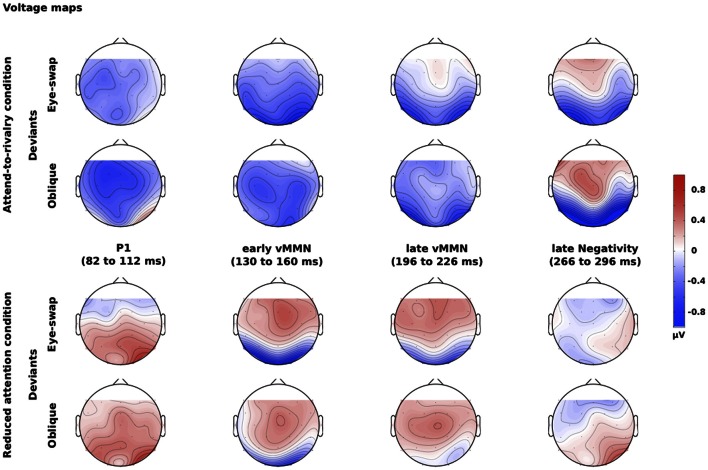
**Voltage maps for the differences between deviants and standards for different times after onset of the stimuli.** The top two rows show the maps for the attend-to-rivalry condition; the bottom two rows show the maps for the reduced-attention condition. The upper of each pair or rows shows the eye-swap deviants; the lower shows the oblique deviants. The columns show the four time windows.

Figure 3 displays voltage maps for the difference waves for both sorts of deviants and for both attention conditions for all four time windows. The voltage maps show that the largest voltages were in the occipital electrodes, which is to be expected for visual stimuli, and that generally the two sorts of deviants yielded similar maps. There were two major differences:

1. There was a reversal of polarity around 100 ms after onset for the reduced-attention condition compared with the other times. This is because there was a deviance-related positivity in this early time window rather than a negativity (see Figure 2).
2. The maps for the reduced-attention condition around 280 ms are rather ill-defined. This is because the deviance-related negativity essentially disappeared in this time window (again, see Figure 2).

We chose four time periods spanning 30 ms each within which we analysed amplitudes for the difference waves shown in Figure 2. The statistical tests we report below confirm our characterization of the results. We tested whether the amplitudes of the difference waves differed from zero using one-tailed *t*-tests; we tested for differences in the difference waves among the various experimental conditions with repeated-measures ANOVAs with factors type of deviant (eye-swap vs. oblique), attention condition (attend-to-rivalry vs. reduced-attention), and hemisphere (left vs. right).

#### 82–112 ms (P1)

One-tailed *t*-tests yielded significant positivities for eye-swap and oblique deviants in the reduced-attention condition at both electrodes [eye-swap deviants: *t*_(13)_ = 2.24, *p* = 0.022 [O1], *t*_(13)_ = 4.31, *p* < 0.001 [O2]; oblique deviants: *t*_(13)_ = 1.78, *p* = 0.05 [O1], *t*_(13)_ = 2.39, *p* = 0.017 [O2]], but not in the attend-to-rivalry condition [eye-swap deviants: *t*_(13)_ = −1.57, *p* = 0.07 [O1], *t*_(13)_ = −0.24, *p* = 0.407 [O2]; oblique deviants: *t*_(13)_ = 0.11, *p* = 0.458 [O1], *t*_(13)_ = 1.34, *p* = 0.099 [O2]]. That is, in the reduced-attention condition, deviants elicited larger positivities than in the attend-to-rivalry condition, *F*_(1, 13)_ = 10.21, *p* = 0.007, partial η^2^ = 0.440.

The positivities presumably arise from adaptation, or “refractoriness” (Kimura, 2012, p. 145): the standards are seen much more often than the deviants, so are processed by adapted neurons, whereas the deviants are rare, so are processed by less-adapted neurons. It is possible the lack of a positivity for the attend-to-rivalry condition comes from a ceiling effect in the ERPs: both standards and deviants yield P1s greater than 2 μV. There is no such ceiling effect in the reduced-attention condition.

#### 130–160 ms (early vMMN)

In the early time window within the first deviance-related negativity we found significant negativities for eye-swap deviants at both occipital electrodes in both conditions [attend-to-rivalry condition: *t*_(13)_ = −3.02, *p* = 0.005 [O1], *t*_(13)_ = −3.49, *p* = 0.002 [O2]; reduced-attention condition: *t*_(13)_ = −3.13, *p* = 0.004 [O1], *t*_(13)_ = −3.51, *p* = 0.002 [O2]]. That is, eye-swap deviants showed a more negative response than standard stimuli whether attention was directed to or withdrawn from the rival gratings. Differences from 0 for the oblique deviants failed to reach significance in the attend-to-rivalry condition [O1: *t*_(13)_ = −1.01, *p* = 0.165; O2: *t*_(13)_ = −1.58, *p* = 0.069] and at the left hemisphere in the reduced-attention condition [O1: *t*_(13)_ = −1.53, *p* = 0.075]. These differences between the two attention conditions failed to reach significance in the ANOVA, *F*_(1, 13)_ = 0.95, *p* = 0.347. In other words, there is a vMMN to both sorts of deviants in the early time window.

#### 196–226 ms (late vMMN)

In the late time window within the first deviance-related negativity we found significant negativies for eye-swap deviants at both occipital electrodes in both conditions [attend-to-rivalry condition: *t*_(13)_ = −1.99, *p* = 0.034 [O1], *t*_(13)_ = −1.99, *p* = 0.034 [O2]; reduced-attention condition: *t*_(13)_ = −2.21, *p* = 0.023 [O1], *t*_(13)_ = −3.50, *p* = 0.002 [O2]]. That is, eye-swap deviants show a more negative response than standard stimuli when attention was directed to or withdrawn from the gratings. All oblique deviants showed negativities, significantly so in the attend-to-rivalry condition at the right hemisphere [O2: *t*_(13)_ = −1.93, *p* < 0.038] but not at the left hemisphere [O1: *t*_(13)_ = −1.73, *p* = 0.054] or in the reduced-attention condition at either hemispheres [O1: *t*_(13)_ = −0.63, *p* = 0.271; O2: *t*_(13)_ = −0.61, *p* = 0.276]. These differences between the two types of deviants failed to reach significance in the ANOVA, *F*_(1, 13)_ = 1.61, *p* = 0.227, again leading us to conclude that similar vMMNs occurred to both sorts of deviants.

#### 266–296 ms (late negativity)

The deviance-related negativity following the P2 component of the ERPs occurs in the attend-to-rivalry condition only, *F*_(1, 13)_ = 9.43, *p* = 0.009, partial η^2^ = 0.420. It is significantly negative for eye-swap and oblique deviants at both occipital electrodes [eye-swap deviants: *t*_(13)_ = −4.00, *p* = 0.001 [O1], *t*_(13)_ = −3.01, *p* = 0.005 [O2]; oblique deviants: *t*_(13)_ = −2.39, *p* = 0.016 [O1], *t*_(13)_ = −2.38, *p* = 0.017 [O2]]. That is, eye-swap and oblique deviants show a more negative response than standard stimuli when attention was directed to the gratings.

## General discussion

We found a deviance-related negativity to eye-swap deviants during binocular rivalry from 140 to 250 ms after onset of the stimuli in both attention conditions and that persisted until about 350 ms when attention was on the rival gratings. We also found similar results for oblique, control deviants. We conclude that this negativity is the vMMN.

We have to admit to at least two limitations on the experimental evidence for our conclusion:

1. The standards, by virtue of being more frequent than deviants, were presumably processed by neurons that are more adapted than those processing deviants. The usual way to overcome this limitation is to equate the frequency of standards and deviants by placing them in sequences in which there are many other sorts of stimuli (Kimura et al., 2009). But there is a practical problem in using this approach with binocular vision—we do not have enough eyes. That is, to equate deviants with a frequency of 20%, one would need to have five eyes! We look forward to future studies in which this issue can be addressed.
2. The oblique deviants differed in at least two ways from the standards: in rareness but also in orientation. That is, we have not tested standards of oblique rival stimuli. We are not too concerned about this because we included the oblique deviants merely to serve as a control condition, from which we would be sure to find a vMMN. We are currently working to unconfound orientation from rareness of rival stimuli. Our preliminary results suggest that there are no differences when rivalry deviants are compared with standards having the same orientations (Jack et al., 2012).

If we can accept that the deviance-related negativity we have found is the vMMN, then there are at least two further conclusions:

1. The vMMN is sensitive to eye of origin. If we do not consider eye of origin, the eye-swap deviants are identical to standards (see Table 1). As far as we are aware, ours is the first demonstration that eye of origin can serve as a source of deviant information that yields a vMMN, and can be added to the other basic properties of visual stimuli, such as orientation, spatial frequency, color, and movement that yield vMMNs (Pazo-Alvarez et al., 2003). That eye of origin can be a basic visual feature is perhaps not surprising when one considers its main function: it is to allow depth perception through stereopsis (Wheatstone, 1838). Swapping the images of a stereogram between the eyes reverses the perceived depth. Of course no stereopsis is possible with our rival stimuli, but this is not to oppose the role of eye of origin in our results. Eye of origin's being a basic, automatically processed feature of visual input is also consistent with other phenomena, such as its also popping out of arrays of stimuli that are being searched (Wolfe and Franzel, 1988).
2. The vMMN is a signature of automatic, low-level processing of regularities and irregularities in input and does not depend on conscious experience, which is presumably mediated by high levels of the visual system and other areas of the brain (e.g., Fries et al., 1997; Gaillard et al., 2009; Lamme, 2010).
  We admit that we cannot prove this conclusion from our results because participants saw (i.e., were conscious of) both sorts of deviants on essentially every trial. This is opposite to what might have been predicted for eye-swap deviants from the findings of Logothetis et al. (1996) and is consistent with the findings of Blake et al. (1980). We cannot rule out that some aspect of the conscious experience of the deviants was responsible for the vMMN to them; we consider this in more detail below.
  Nevertheless, there is abundant evidence for low-level processing of regularities and irregularities from other studies than ours both for visual input (e.g., Czigler, 2007) and for auditory input (the MMN; e.g., Sussman, 2007; Sadia et al., 2013), but we like to think that binocular rivalry presents a stringent test of this in that its experience is unpredictable (Fox and Herrmann, 1967; Levelt, 1967; Zhou et al., 2004). It is also consistent with the electrodes from which we found the vMMN—occipital electrodes over the visual areas of the brain—and with the early time of ERP differences in response to changes to one of the rival stimuli of which participants are either aware or not (Roeber and Schröger, 2004; Roeber et al., 2008, 2011; Veser et al., 2008). It is also consistent with our finding a vMMN in the reduced-attention condition; the 2-back task was so demanding that participants either missed seeing most of the changes in orientation of the gratings or missed seeing all of them.
  We have painted low and high levels with a rather broad brush. It is quite possible that there are levels within those levels at which the comparisons between some model of regularities in visual input and the visual input to a lower level are made (Garrido et al., 2009). Our point is that these lower levels really are low—close to the neurons in the visual cortex that first combine the inputs from the left eye and right eye, because these are the first neurons that can encode eye of origin.

**Table 1:**
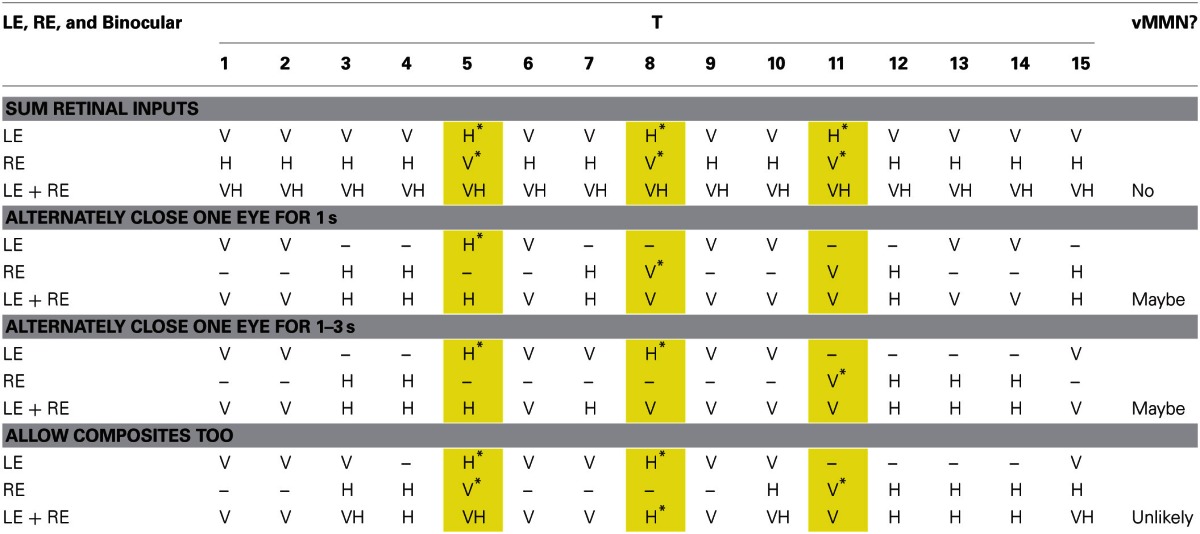
**Possible sequences of 15 stimuli (standards and deviants) and percepts that are closer and closer approximations to the experience of rivalry**.

As we said we cannot rule out that some aspect of the experience of deviants yields the vMMN because the participants experienced the deviants in the attend-to-rivalry condition. We are conducting other research with deviants that are presented to only one eye during binocular rivalry (Roeber et al., submitted). Our preliminary results suggest that vMMNs can be evoked by deviants that are invisible because of rivalry suppression. But we can rule out, in the current study that a participant could figure out the rule that defines a deviant from his or her experience of orientations in the attend-to-rivalry condition, because that experience is unpredictable. To understand this, we have illustrated in Table 1 some examples of sequences of experienced orientations from what rivalry is *not*.

In Table 1, we show 15 presentations of the stimuli, from left to right (i.e., T1, T2, and so on). We show four cases, each one representing a successively closer approximation of the experience of binocular rivalry. For each case, we show what consciousness would be like if it were contributed to only by the left eye (LE), only by the right eye (RE) and as if binocular vision simply summed up the inputs from the LE and RE. The orientations are coded as V for vertical, and H for horizontal. We show three eye-swap deviants in the yellow columns. We give an asterisk if a deviant could possibly be experienced as a deviant.

In the first case, we show what would happen if consciousness consisted of simply summing the input from the LE alone and from the RE alone. Note that each eye alone yields three clear deviants, but that with both eyes open, there are no deviants. We know from EOG electrodes that all participants kept both eyes open for all accepted epochs, so this case demonstrates that the vMMN must arise from eye-of-origin information. We also know that binocular vision did *not* sum the LE and RE input; rather there was binocular rivalry.

In the second case, we show what would happen if rivalry were like a participant's alternately winking one or the other eye for 1 s each. In each eye, this yields pairs of presentations of gratings (i.e., two 400-ms presentations plus two 100-ms ITIs) interspersed by pairs of presentations of darkness. Again each eye alone could generate a vMMN, but both eyes do not reveal any clear deviants (although it is possible over longer sequences there could be some rules that could identify deviants). But again, we know that binocular rivalry is *not* like alternately winking the eyes at a regular rate.

In the third case, we show what would happen if rivalry were like a participant's alternately winking one or the other eye for a random time from 1 to 3 s (this temporal sequence is more like that of a typical experience of rival images than the second case). Again each eye alone could generate a vMMN, but both eyes do not reveal any clear deviants (although it is possible over longer sequences there could be some rules that could identify deviants). But again, we know that binocular rivalry is *not* like randomly, alternately winking the eyes.

In the fourth case, we show what would happen if rivalry were like the third case, except that at transitions from one percept to the next, participants saw composites of the images from each eye. All of this makes the experience of rivalry unpredictable, ruling out any vMMNs being developed to experience of both eyes.

A further complication is that visibility of a stimulus from one eye during rivalry is *not* as we have represented it—that is it like one eye is closed—but it is simply an attenuation of visibility (Fox and Check, 1966; Alais et al., 2010). Moreover, composites are neither simple nor stable—they are complex, representing superimpositions or patchworks, and they are dynamic. All of this should serve to make the experience of rivalry completely unpredictable and to prevent any regularities from being extracted against which to contrast deviants.

In conclusion, our study is a first step on a journey to prove that eye of origin can serve as a deviant that will yield a vMMN and to combine the fields of research into binocular rivalry and into processing of regularities in visual input. We look forward to our and others' taking further steps on this journey.

### Conflict of interest statement

The authors declare that the research was conducted in the absence of any commercial or financial relationships that could be construed as a potential conflict of interest.
